# Global transmission and distribution of phage-encoded cholera toxin genes constrained by toxin-repression genes and anti-phage defense systems

**DOI:** 10.1093/ismejo/wrag139

**Published:** 2026-06-11

**Authors:** Shujian Yuan, Demeng Tan, Dong Zhu, Jose Luis Balcazar, Hui Wang, Ville-Petri Friman, Mingming Sun, Feng Hu

**Affiliations:** Soil Ecology Lab, Jiangsu Provincial Key Laboratory of Coastal Saline Soil Resources Utilization and Ecological Conservation, Jiangsu Collaborative Innovation Center for Solid Organic Waste Resource Utilization & Jiangsu Key Laboratory for Solid Organic Waste Utilization, Nanjing Agricultural University, Nanjing 211800, China; Shanghai Public Health Clinical Center, Fudan University, Shanghai 201508, China; Zhejiang Key Laboratory of Urban Environmental Processes and Pollution Control, Ningbo Urban Environment Observation and Research Station, Chinese Academy of Sciences, Ningbo 315800, China; Catalan Institute for Water Research (ICRA), Girona 17003, Spain; Department of Microbiology, Nanjing Agricultural University, Nanjing 210095, China; Department of Microbiology, University of Helsinki, Helsinki 00014, Finland; Soil Ecology Lab, Jiangsu Provincial Key Laboratory of Coastal Saline Soil Resources Utilization and Ecological Conservation, Jiangsu Collaborative Innovation Center for Solid Organic Waste Resource Utilization & Jiangsu Key Laboratory for Solid Organic Waste Utilization, Nanjing Agricultural University, Nanjing 211800, China; Soil Ecology Lab, Jiangsu Provincial Key Laboratory of Coastal Saline Soil Resources Utilization and Ecological Conservation, Jiangsu Collaborative Innovation Center for Solid Organic Waste Resource Utilization & Jiangsu Key Laboratory for Solid Organic Waste Utilization, Nanjing Agricultural University, Nanjing 211800, China

**Keywords:** cholera, toxin genes, phage, horizontal gene transfer, toxin-repression genes

## Abstract

Cholera is a severe diarrheal disease caused by toxigenic *Vibrio cholerae*, whose virulence depends on lysogenic infection by CTXφ bacteriophages encoding the cholera toxin genes (*ctxA* and *ctxB*) and associated accessory genes (*ace* and *zot*). However, the global distribution and transmission dynamics of phage-encoded cholera toxin genes across environments remain poorly understood. To address this, we performed a large-scale bioinformatic analysis of publicly available whole genomes. We show that both phages and bacteria carrying toxin genes are globally distributed across human-associated, freshwater, fish, and mammalian habitats, with *Vibrio* and *Aeromonas* being the dominant bacterial taxa and *Inoviridae* is the most prevalent phage family. Phage-mediated horizontal gene transfer (HGT) of toxin genes occurred in both *Vibrio* and non-*Vibrio* species, with the highest transfer between *Inoviridae* and *V. cholerae* occuring predominantly among bacteria from the same habitat. Temporal analysis revealed an increase in candidate HGT events after 2000, peaking at 377845 events during 2010–2019. HGT events negatively correlated with the presence of CRISPR-Cas system and toxin-repression genes (*hns, hapR*, and *tsrA*) in host bacteria. Experimental validation indicated that H-NS and HapR inhibit phage infection by repressing phage release. Together, our results suggest that CRISPR-Cas phage defense system and toxin-repression mechanisms could constrain the spread of toxin-carrying phages, with potential implications for the occurrence and severity of cholera outbreaks worldwide.

## Introduction

Cholera is an acute diarrheal disease caused by infection with pathogenic *Vibrio cholerae* serogroups O1 or O139 [1] and continues to pose a major global public health threat, particularly in regions with limited access to clean water and sanitation [2]. The primary virulence factor of cholera, cholera toxin (CT), is encoded by the *ctxAB* operon carried on the lysogenic filamentous bacteriophage CTXφ integrated into *V. cholerae* genomes [3]. Although *ctxAB*, together with the accessory genes *ace* and *zot*, are classically associated with toxigenic *V. cholerae* strains [4], the evolutionary context of CT extends beyond a single bacterial lineage [5]*.* CT belongs to the broader AB_5_ toxin family, which also includes the heat-labile enterotoxins (LT) produced by *Escherichia coli*, sharing substantial structural and sequence similarity [6–8]. This relationship suggests a shared evolutionary origin and highlights the potential mobility of toxin modules across bacterial taxa. Homologous *ctxAB* genes have also been identified in *Vibrio mimicus*, where they contribute to pathogenicity in human infections [9, 10]. In addition, fragments of CTXφ-associated genes, including *ace* and *zot*, have been detected in environmental isolates of genera such as *Aeromonas* and *Enterobacter*, indicating that components of the CTXφ genetic repertoire may circulate among diverse aquatic bacteria [11]. Despite these observations, the precise phylogenetic relationships between *Vibrio*-derived toxins and homologous sequences in non-*Vibrio* hosts remain poorly resolved. It is therefore unclear whether these sequences represent deeply diverged toxin lineages or more recent horizontal gene transfer (HGT) events mediated by filamentous phages such as VGJφ [12].

The apparent constraint of high-level CT expression and stable maintenance within pathogenic *V. cholerae* suggests that host-specific factors regulate the successful acquisition of these genes [13]. Bacteria have evolved a multi-layered defense suite to limit such phage-mediated HGT, including restriction-modification systems, CRISPR-Cas adaptive immunity, and the *avcD* defense system among others [14–17]. In *Vibrio cholerae*, these systems are complemented by global transcriptional regulators that modulate expression of horizontally acquired virulence genes, including the CTXφ-encoded CT and the toxin-coregulated pilus (TCP) [18–20]. Key nucleoid-associated proteins such as H-NS and TsrA act predominantly as repressors of virulence gene expression, particularly silencing genomic islands and phage-associated loci, whereas the quorum-sensing (QS) regulator HapR downregulates virulence functions at high cell density [21]. Through integration of environmental cues, population density signals, and intrinsic chromatin structure, these global regulators balance toxin production in other pathogenic traits [22]. Despite these insights into defense systems and transcriptional control, it remains unclear how they collectively influence the global distribution of CT genes across bacterial clades and their mobility across species boundaries.

Here, we combined large-scale bioinformatics analyses with experimental validation to investigate the global ecology of phage-encoded cholera toxins. We compiled bacterial and phage genome sequences encoding *ctxA, ctxB, ace,* and *zot* from RefSeq, EnteroBase, and IMG/VR databases to map their distribution across species, habitats, and decades. Using comparative genomics and generalized linear mixed models, we assessed how bacterial toxin-repression genes and phage defense systems influence phage-mediated HGT across habitats and through time. Our analyses revealed that horizontal transfer of toxin genes occurs more frequently within shared habitats than across distinct environments and has increased in the twenty-first century, with a notable surge between 2010 and 2020. Both bacterial toxin-repression genes (*hns, hapR*, and *tsrA*) and phage defense systems (CRISPR-Cas system) were negatively correlated with HGT events. Experimental validation using *V. cholerae* demonstrated that H-NS and HapR limit phage-mediated toxin transfer by inhibiting phage release. Collectively, our findings suggest that the distribution and activity of CT genes are governed by a co-evolutionary arms race between phages and bacteria, wherein bacterial defense and toxin-repression systems constrain the mobility and expression of phage-encoded toxins, with implications for the dynamics of cholera epidemics.

## Materials and methods

### Collection of bacterial genomes encoding toxin genes

The toxin gene sequences (*ctxA, ctxB, ace*, and *zot*) were used to search against comprehensive bacterial genomes in the RefSeq and EnteroBase databases. Initial candidates were identified using BLASTp with a trusted cutoff (*e*-value <1e^−5^, Identity = 40.0%). To minimize false positives and confirm functional potential, all BLAST-identified toxin genes were subjected to a Hidden Markov Model (HMM) search. We utilized HMMER (v3.3.2) to identify specific functional domains for *ctxA, ctxB, ace*, and *zot* based on Pfam database profiles. Only sequences that showed positive matches in both the BLAST similarity search and the HMM domain search were retained for further analysis. To exclude distant homologues that might not function like true cholera toxins, we performed a maximum-likelihood phylogenetic analysis using IQ-TREE (Figs S1-S4). Sequences identified from both *Vibrio* and non-*Vibrio* species were aligned with reference sequences from known *V. cholerae* strains. Only those candidates that clustered robustly within the monophyletic clade defined by the *V. cholerae* reference toxins were confirmed. This multi-step validation identified a final set of 2004 bacterial genomes encoding validated toxin genes.

### Identification of viral sequences and prophages

To ensure comprehensive capture of viral sequences and minimize false negatives, we combined de novo prophage prediction with established viral databases. We applied a dual prophage detection strategy using geNomad and PhiSpy to identify prophage regions within the toxin-carrying bacterial genomes, leveraging complementary strengths of each tool to improve detection of diverse prophages. These predicted prophage sequences were merged with phage sequences retrieved from the IMG/VR database (v4.1), which includes both free and temperate phages with thorough annotation quality control. Following the same three-stage validation pipeline described above (BLAST similarity, HMM domain search, phylogenetic clustering), we obtained a final non-redundant dataset comprising 1747 phage sequences with validated CT genes. Integrase families of phages were annotated by searching against Pfam profiles for Tyrosine Integrases and Serine Integrases.

### Collection of toxin-repression factors and mobile genetic elements

Consistent with previous studies that bacteria may encode genes to repress CT gene expression or limit acquisition of exogenous genes [21, 23], we collected known toxin-repression factors, including *hns, hapR*, and *tsrA* from the NCBI database, and phage resistance systems (*avcD* and restriction–modification systems) from KEGG. These genes were identified using BLASTp with a trusted cutoff (*e*-value <1e^−5^ and ≥ 40.0% amino-acid identity). The CRISPR–Cas system was identified using CRISPRCasTyper (v1.8.0) to detect CRISPR arrays and associated Cas genes in bacterial genomes [24]. These genes and systems were collectively defined as toxin-repression factors. Mobile genetic elements (MGEs) in bacterial genomes were identified using MobileElementFinder (v1.1.2).

### Global distribution of cholera-associated bacterial and phage sequences

**Figure 1:**
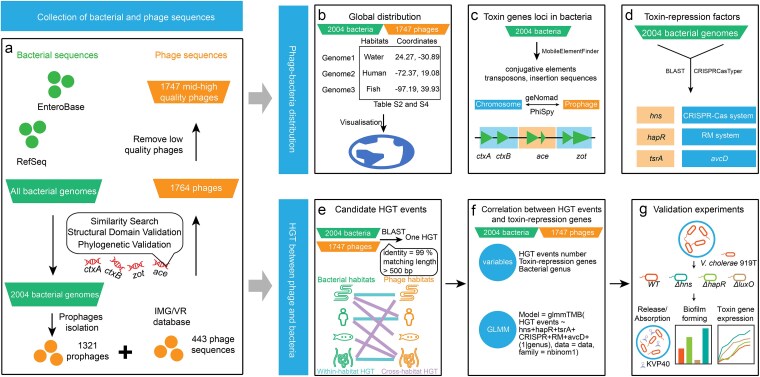
Overview of analysis steps. a, Collection of bacterial and phage genomes carrying toxin genes (*ctxA, ctxB, ace*, and *zot*) from public datasets (RefSeq, EnteroBase, IMG/VR databases). b, Geographic and habitat distribution of toxin-encoding bacterial and phages genomes based on metadata from public datasets. c, Identification of mobile genetic elements (MGEs; including integrative and conjugative elements, transposons, insertional sequences, and prophages) in bacterial genomes using MobileElementFinder, geNomad, and PhiSpy. d, Identification of toxin-repression factors, including CRISPR-Cas system (CRISPRCasTyper), and toxin-repression factors (including *hns, hapR, tsrA, avcD*, and RM system (BLASTp)). e, Calculation of the toxin genes HGT events between bacterial and phage genomes. BLASTn (99.0% similarity and matching length > 500 bp) was used to match the DNA blocks shared by bacterial genomes and phages. Phage-mediated HGT of toxin genes was compared within and across different habitats. f, Generalized linear mixed-effects models (GLMMs) were applied to evaluate the effects of anti-phage defense systems (*avcD*, CRISPR–Cas, and RM systems) and toxin-repression genes (*hns, hapR*, and *tsrA*) on phage-mediated HGT events involving toxin genes. g, The *hapR, luxO*, and *hns* were knocked out in *Vibrio cholerae* 919T to verify the effect of the toxin-repression genes on KVP40 phage release, adsorption, the biofilm formation, and toxin gene expression of *V. cholerae* 919T.

The global distribution of bacterial strains was determined using biosample information from the RefSeq database and the geographic location and sampling time provided by the EnteroBase database (Fig. 1; Table S1). Geographic coordinates of phages carrying CT genes (*ctxA, ctxB, ace*, and *zot*) were obtained from the IMG/VR database along with habitat information (Fig. 1; Table S2) [25]. Sequences originating from the same country were assigned a single representative coordinate. We integrated epidemiological data on reported cholera cases from 1949 to 2021 obtained from Our World in Data (https://ourworldindata.org/grapher/number-reported-cases-of-cholera). For each country *i*, the case weights of global reported cases were calculated:


(1) \begin{eqnarray*} {P}_i=\frac{C_i}{\sum_{j=1}^n{C}_j} \end{eqnarray*}

where *C_i_* represents the total number of reported cholera cases in country *i*, and ∑C*_j_* represents the global total reported cholera cases across all countries included in the dataset. This case-based weighting *P_i_* and the number of genomes were used to calculate a case-weighted genomic abundance metric [26]:


(2) \begin{eqnarray*} {W}_i={G}_i\times{P}_i \end{eqnarray*}

where G*_i_* is the number of bacterial or phage genomes harboring toxin genes from country *i* and 𝑊*_i_* is the resulting case-weighted genomic abundance metric. The case-weighted genomic abundance metric is designed to partially correct for geographical sampling bias in publicly available genomic databases. This metric estimates the relative expected detection levels of toxin-encoded bacteria across countries under a scenario in which sequencing effort is proportional to cholera burden. It was used for comparisons between countries rather than to indicate actual environmental abundance of microbes. Rarefaction-based methods were not employed because they rely on the observed genomic dataset, require a minimum level of genomic sampling and cannot reasonably estimate representation in severely under-sampled or completely unsampled countries. The case-weighted method is less sensitive to missing genomic surveillance data and can adjust the weights for countries with severely mismatched cholera burden and sequencing efforts, providing an epidemiologically informed correction for geographic sampling bias. Metadata on bacterial and phage habitats were extracted from the associated sequence annotations. Geographic distributions were visualized using the ggplot2 package in R.

### Candidate HGT event analysis between phages and bacteria

HGT between phages and bacteria was analysed using sequence similarity searches. Shared DNA fragments between phage and bacterial genomes were identified using BLASTn (v2.13.0+). Alignments showing ≥99.0% nucleotide identity and ≥ 500 bp of shared sequence were considered HGT signals (Fig. 1). To avoid biases introduced by differences in genome size or the number of alignments, raw BLAST hits were not used directly to quantify HGT. Instead, following previously described approaches, candidate HGT events were defined as the number of genome pairs sharing at least one HGT signal. This strategy was applied in three contexts: (i) phage–bacteria comparisons across habitats; (ii) interspecies comparisons among bacterial genomes [27, 28]; (iii) phage–bacteria comparisons across decades. This sequence similarity framework alone does not constitute definitive evidence of HGT. Sequence similarity may arise through multiple evolutionary processes, including shared ancestry, recombination, or other forms of sequence conservation. The HGT signals reported here were thus considered as ``candidate HGT events''.

### Effect of toxin-repression factors on the HGT of toxin genes

The influence of toxin-repression factors on the HGT events between phages and bacteria was assessed using a Generalized Linear Mixed Model (GLMM), in which the intercept of each anti-phage defense factor was allowed to vary [28]. Bacterial genus was included to be a random effect to account for genus-specific differences in HGT dynamics (Fig. 1). Models were fitted using the *glmmTMB* function in R, and *P* values for toxin-repression factors were obtained via likelihood ratio tests. Model performance and stability were evaluated using Nakagawa’s *R*^2^. To further assess stability, overdispersion and outliers were tested with the DHARMa package, and the root mean squared error (RMSE) with standard deviation (SD) was calculated using five-fold cross-validation. For each bacterial genome, the number of toxin-repression genes (*hapR, hns*, and *tsrA*) and anti-phage defense systems (*avcD*, CRISPR-Cas, RM system) was determined. The incidence rate ratio (IRR) of each toxin-repression gene and anti-phage defense system was then estimated from the GLMM to quantify their effect on HGT counts across bacterial genera. In this analysis, HGT events were defined as the number of detected HGT events per genome, and the variable genus was included with a random effect.

The following GLMMs were fitted to HGT counts derived from DNA blocks ≥500 bp and ≥ 99.0% nucleotide identity: Model 1 included all toxin-repression genes and anti-phage defense systems were fixed effects with genus were a random intercept, Model 2 assessed the effect of *hns* with a random slope by genus, Model 3 assessed *hapR* with a random slope, and Model 4 assessed *tsrA* with a random slope. The models were listed follows:

Model 1: glmmTMB(HGTevents ~ *hns* + *hapR* + *tsrA* + CRISPR-Cas + RM + *avcD* + (1 | genus), data = data, family = nbinom1).

Model 2: glmmTMB(HGTevents ~ *hns* + (0 + *hns* | genus), data = data, family = nbinom1).

Model 3: glmmTMB(HGTevents ~ *hapR* + (0 + *hapR* | genus), data = data, family = nbinom1).

Model 4: glmmTMB(HGTevents ~ *tsrA* + (0 + *tsrA* | genus), data = data, family = nbinom1).

The contribution of individual toxin-repressing genes to HGT events across bacterial genera was evaluated using analysis of variance (ANOVA). For example, the effect of *hns* was tested by comparing a random-slope model (Model 2) with a fixed-effect-only model (Model 2.1 = glmmTMB(HGTevents ~ *hns*, data = data, family = nbinom1)) using a likelihood ratio test (anova(Model 2, Model 2.1, test = “Chisq”)).

### Construction of Δ*hns* and QS (Δ*hapR* and Δ*luxO*) mutant strains

Three *V. cholerae* 919 T mutant strains (Δ*hns*, Δ*hapR*, and Δ*luxO)* were constructed to examine the role of toxin-repression and QS regulators in the acquisition of exogenous genes (Fig. 1). The bacterial strains, phages, and plasmids used in this study are detailed in Table S3. Each mutant was generated by deleting the corresponding gene (*hns, hapR,* or *luxO*) from the chromosome. *V. cholerae* 919 T was cultured aerobically at 37°C in LB Miller broth or on LB agar (1.5%). Chromosomal deletions were generated using a homologous recombination strategy like previously described [29]. Briefly, the upstream and downstream flanking regions of each target gene were amplified using primers listed in Table S4. The resulting fragments, sharing ~30-bp overlapping sequences, were used as templates for a second polymerase chain reaction (PCR) to generate deletion constructs. The assembled PCR products were digested with appropriate restriction enzymes and cloned into the suicide plasmid pDM4.

The recombinant pDM4 constructs were introduced into *V. cholerae* 919 T by conjugation using *Escherichia coli* S17–1 (the donor strain) at a donor:recipient ratio of 10:1. Overnight cultures of both strains were diluted 1:100 in LB medium and grown at 37°C until reaching an OD_600_ of ~1.0. Donor and recipient cultures were mixed and plated on LB agar, followed by overnight incubation at 37°C to allow conjugation. Transconjugants were selected on thiosulfate–citrate–bile salts–sucrose (TCBS) agar supplemented with chloramphenicol (5 μg ml^−1^) [30]. Individual colonies were purified on LB agar and subsequently screened for plasmid excision by growth on nutrient-limited agar (5 g L^−1^ peptone, 1 g L^−1^ yeast extract, and 5 g L^−1^ NaCl) containing 5.0% sucrose for 48 h at room temperature. Colonies that lost chloramphenicol resistance were screened by PCR, and the resulting deletion mutants were confirmed by Sanger sequencing.

### Effects of WT, Δ*hns*, and QS mutants on phage release and adsorption

To evaluate the influence of *V. cholerae* wild-type (WT) 919 T, Δ*hns*, and QS mutants (Δ*hapR* and Δ*luxO*) on phage KVP40 replication and release, we measured phage proliferation and adsorption (Fig. 1). Phage lytic activity was assessed at a multiplicity of infection of ~0.0001–0.0002 in LB medium, and phage concentrations were determined at regular intervals using plaque assays. Phage adsorption rates were quantified by monitoring the decline of free phages in the culture supernatant during infection. Cultures were incubated at 37°C with constant shaking (200 rpm), and supernatant samples were collected every 4 minutes. Phage concentrations in the supernatant were determined via the double-layer agar plaque method as described previously [31]. Adsorption rates was calculated using the formula [31]:


(3) \begin{eqnarray*} Adsorption\ rates=\frac{\ln{P}_0-\ln{P}_t}{B_0} \end{eqnarray*}

where *P_0_* and *B_0_* are the numbers of phages and bacteria added at *t* = 0, and *P_t_* is the concentration of free phage particles in the supernatant at time *t*. This analysis enabled direct comparison of phage binding efficiency among the WT and mutant strains.

### Growth, biofilm, and plasmid conjugation efficiency quantification

*V. cholerae* 919 T, Δ*hns*, and QS mutants (Δ*hapR* and Δ*luxO)* were grown overnight in LB at 37°C [32, 33]. Pre-cultures were diluted 1:1000 and inoculated into either LB broth or sea salt buffer (Sigma-Aldrich, St. Louis, MO, USA) supplemented with 10.0% LB to a final volume of 15 ml. Growth was monitored by measuring optical density at 600 nm (OD_600_) at 1-h intervals for 8 h [32]. Biofilm formation was assessed using a crystal violet (CV) staining assay [34]. Briefly, overnight cultures were diluted 1:1000 and inoculated into 5 mL LB in polystyrene tubes, and incubated for 10 days to allow biofilm formation. After incubation, the supernatant was removed, and the tubes were washed with SM buffer. Biofilms were stained with 6 ml of 0.4% CV solution (Sangon, Shanghai, China) for 15 min, then washed with SM buffer to remove excess dye and air-dried for 5 min. Bound CV was solubilized by adding 6 ml of 33.0% acetic acid and incubating for 5 min. Absorbance of the solubilized CV was measured at 595 nm to quantify biofilm formation. To assess the effect of studied mutations on conjugation efficiency, donor strain *E. coli* DH5α carrying plasmid pHB20TG was used, while recipient strains consisted of *V. cholerae* WT 919 T, and its Δ*hns* and QS mutants (Δ*hapR* and Δ*luxO*). The overnight strains underwent dilution and were adjusted to an OD of 1.0. The donor and recipient were washed in LB medium twice and mixed in equal volumes. Subsequently, 30 μl aliquots were placed on LB agar plates and incubated at 37°C for 6 h. After 6 h of incubation, the bacterial mixtures were scraped and resuspended in PBS buffer. Serial dilutions were plated onto TCBS plates, with or without gentamicin (15 μg ml^−1^), and then incubated at 37°C in order to count colony-forming units. The relative conjugation efficiency was calculated from the ratio of the transconjugants to the total number of colonies of the WT strain 919 T, Δ*hns* and its QS mutants (Δ*hapR* and Δ*luxO*).

### Real-time quantitative PCR

*Vibrio cholerae* WT, Δ*hns*, and QS (Δ*hapR* and Δ*luxO*) were grown overnight in LB broth. Pre-cultures were diluted 1:10 000 in fresh LB and incubated at 37°C for 6 h. Aliquots were then collected for RNA extraction targeting *ctxA, ctxB*, and *recA* transcripts. Total RNA was isolated using the TRIzol reagent following the manufacturer’s instructions (Thermo Fisher Scientific). Residual genomic DNA was removed by DNase I treatment, and RNA was inactivated with ethylenediaminetetraacetic acid (EDTA) at 80°C for 5 min. DNA-free RNA samples were stored at −80°C until cDNA synthesis. First-strand cDNA was generated from 0.5 μg RNA using the RevertAid First-Strand cDNA Synthesis Kit (Thermo Fisher Scientific) according to the manufacturer’s protocol. Gene expression levels of *ctxA* and *ctxB* were quantified relative to *recA* using SsoAdvanced SYBR Green Supermix (Bio-Rad) following established qPCR procedures. The comparative threshold cycle (CT) method was used for relative quantification of RNA [35].

### Transmission electron microscopy of *V. cholerae* strains and phage KVP40

Negative-stain transmission electron microscopy was performed to visualize *Vibrio cholerae* WT, Δ*hns*, and QS mutants (Δ*hapR* and Δ*luxO*), as well as phage KVP40 [34, 36]. Overnight bacterial cultures were diluted 1000-fold in fresh LB broth and grown to the exponential phase. Cells were then fixed with 2.5% glutaraldehyde (Sangon, Shanghai, China). For transmission electron microscopy (TEM) preparation, 20 μl of the fixed culture was deposited onto 200-mesh formvar/carbon-coated copper grids and incubated for 10 min at room temperature. The grids were subsequently negatively stained with 2.0% phosphotungstic acid (SPI-Chem, USA) for 3 min, and excess stain was removed with filter paper. The grids were air-dried for 10 min and examined using an HT7800 transmission electron microscope (Hitachi, Tokyo, Japan).

## Results

### Geographic distribution of bacteria and phages that encode CTgenes

The global distribution of toxin-encoding bacteria across different habitats was investigated to obtain a comprehensive view of the bacterial hosts and prophages associated with CTgenes. We recovered 2004 bacterial genomes carrying CTgenes (*ctxA, ctxB, ace*, and *zot*) from the EnteroBase and RefSeq databases. Across the analyzed genomes, the vast majority (91.0%) were assigned to the genus *Vibrio*, with *Aeromonas* (7.6%), and *Klebsiella* (0.7%) representing the next most frequent taxa; the remaining 10 genera occurred at much lower prevalence (Table S1). To mitigate geographic sampling bias in public genomic repositories, we applied a case-weighted genomic abundance approach that integrates epidemiological data on reported cholera incidence. Specifically, observed genome counts from each country were weighted according to that country’s proportion of global reported cholera cases. This approach increases the relative contribution of genomes originating from regions with a high disease burden but limited sequencing effort, thereby providing a more epidemiologically informed estimate of global cholera distribution.

**Figure 2:**
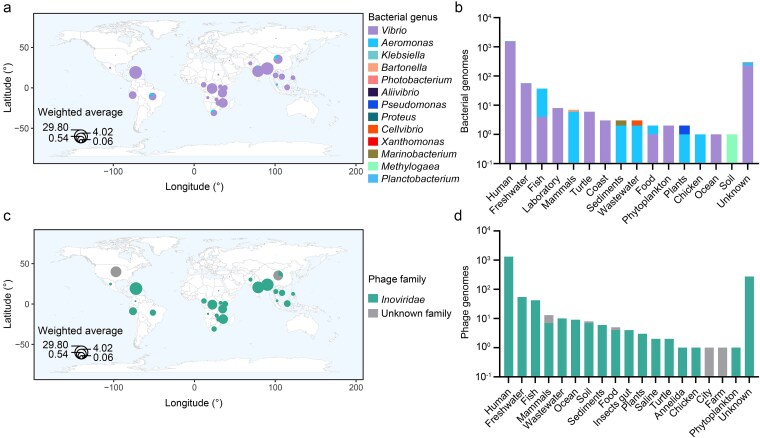
Global spatial and ecological distribution of bacterial genomes and phages encoding cholera toxin genes (*ctxA, ctxB, ace*, and *zot*). a. Geographic distribution of bacterial genomes (*Vibrio* and non-*Vibrio*) carrying the queried toxin genes, sourced from RefSeq and EnteroBase database. Circle size is proportional to the case-weighted bacterial genomic abundance per country (weighted by national cholera case burden); bacterial sequences from the same country are collapsed to a single representative coordinate. b, Stacked bar plot showing the numbers of bacterial genera (as in A) across ecological habitats or isolation sources. c, Geographic distribution of phages carrying the toxin genes. Circle size reflects case-weighted phage genomic abundance (by cholera case burden). d, Stacked bar plot showing the numbers of phage families across ecological habitats or isolation sources.

Under this weighted framework, Haiti and Bangladesh were the dominant hotspots, with case-weighted genomic abundances of 25.1 and 20.6, respectively, far exceeding those of other countries (Fig. 2a). India and the Democratic Republic of the Congo formed a second tier with substantial case-weighted genomic abundances of 12.3 and 6.3, respectively. Overall, the highest weighted abundance of toxin-containing bacterial genomes was concentrated in South Asia (Bangladesh and India), the Caribbean (Haiti), and sub-Saharan Africa (Democratic Republic of the Congo, Mozambique, and Tanzania). The substantial discrepancies were observed between observed and weighted genome counts, reflecting uneven sequencing effort across regions. For example, countries such as the United States (71 observed vs. 0.004 case-weighted) and the United Kingdom (94 observed vs. 0.006 case-weighted) exhibited relatively high sequencing representation despite low cholera incidence, whereas several high-burden countries showed the opposite pattern.

Analysis of habitat metadata indicated that human hosts, freshwater environments, and fish were the primary habitats associated with toxin-encoding bacterial genomes. Human- and freshwater-derived genomes were predominantly affiliated with *Vibrio*, whereas fish-associated genomes were largely dominated by *Aeromonas* (Fig. 2b). These findings indicate that toxin-containing bacteria exhibit a broad geographic distribution and are primarily associated with human hosts, although they are also present in environmental reservoirs such as freshwater and aquatic animals. We next investigated whether these toxin genes were located within prophage regions of bacterial genomes. In total, 1321 toxin-containing prophages were identified, the majority of which belonged to the family *Inoviridae* (Table S2). Overall, 61.6% of bacterial toxin genes were located within identified prophage regions (Table S5). To further assess the mobility potential of these genes, we searched for additional MGEs, including integrative and conjugative elements, transposons, and insertion sequences, within the 10-kb flanking regions of each toxin gene. Approximately 6.9% of toxin genes were associated with MGEs, and in total, 69.3% of toxin genes were located within prophage or MGE regions, indicating a high potential for HGT (Table S5).

Given the large proportion of toxin genes associated with prophages, we further investigated the distribution of toxin-encoding phages, including prophages recovered from bacterial genomes and phage sequences obtained from the IMG/VR database. In total, 1747 toxin-encoding phages were identified (1321 prophages and 426 phage sequences; Tables S2 and S6). Of these, 98.9% were classified within the *Inoviridae* family, whereas the remaining sequences belonged to unclassified viral lineages within the class Caudoviricetes. Similar to the bacterial genomes, phage counts were adjusted using the case-weighted approach based on country-level cholera incidence. This analysis again identified Haiti, Bangladesh, and India as the dominant global hotspots, with case-weighted genomic abandances of 23.3, 18.8, and 13.6, respectively (Fig. 2c). The Democratic Republic of the Congo and Mozambique formed a secondary tier with substantial weighted counts of 4.0 and 3.6. Habitat metadata further indicated that human-associated samples accounted for 74.9% of toxin-encoding phages, followed by freshwater (3.1%) and fish (2.4%) (Fig. 2d). These findings indicate that toxin-containing bacteria and phages exhibit a broad geographic distribution and are primarily associated with human hosts, although they are also present in environmental reservoirs such as freshwater and aquatic animals.

## Phage-mediated horizontal transfer of toxin genes across bacterial species and decades

**Figure 3:**
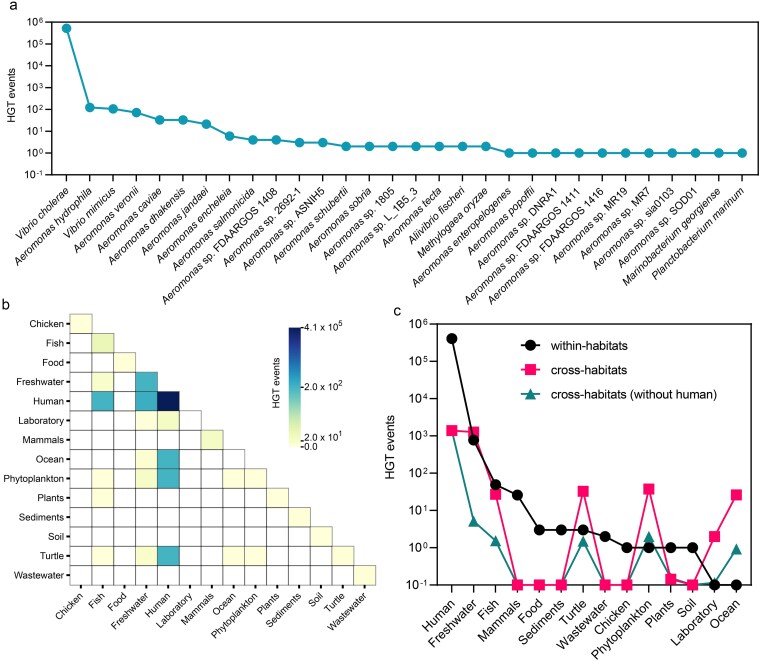
Horizontal gene transfer (HGT) events of cholera toxin genes between phages and bacteria. a, HGT events of cholera toxin genes between bacterial genera and members of the *Inoviridae* family, ranked from highest to lowest. b, Heatmap showing average HGT events of cholera toxin genes between bacterial hosts and phages across different source habitats. c, Average HGT events of cholera toxin genes between same (Within-habitats) and diffetent habitats (Cross-Habitats). Circles: HGT events within-habitats; squares: HGT events crosss-habitats; triangles: HGT events crosss-habitats (excluding human-associated habitats). Points represent observed numbers of HGT events; lines connect values for visual comparison.

We compared sequence similarity between phage and bacterial genomes to investigate phage-mediated HGT of toxin genes. Candidate HGT events were defined as the presence of DNA segments ≥500 bp exhibiting 99.0% sequence identity between phage and bacterial genomes [27]. Because for example shared ancestry or recombination could also explain sequencey similarity, the HGT signals reported here should be considered as “candidate HGT events”. However, for brevity these “candidate HGT events” are simply referred as HGT events from here on. Using this approach, toxin-associated HGT events were detected between 1681 phage genomes and 1778 bacterial genomes. These interactions involved a single phage family, *Inoviridae*, and six bacterial genera: *Vibrio, Aeromonas, Alivibrio, Marinobacterium, Methylogaea*, and *Planctobacterium* (Fig. 3a). Among these, the highest HGT events was observed between *Inoviridae* phages and *V. cholerae* (524479), whereas substantially lower HGT events were detected for *Inoviridae* with *Aeromonas hydrophila* (123) and *V. mimicus* (106) (Fig. 3a). Temporal analysis further revealed variation in phage-mediated HGT through time (Fig. S5). From 1940 to 1999, HGT events remained consistently low (<10 000). In contrast, a marked increase was observed in the twenty-first century, with HGT events rising sharply and peaking at 377845 during 2010–2019. Collectively, these results indicate that phage-mediated transfer of toxin genes occurs predominantly between *Inoviridae* phages and *V. cholerae*, with a pronounced increase in HGT events observed in recent decades.

## Phage-mediated HGT events of toxin genes is elevated within the same habitats

Phage-mediated HGT events of toxin genes was compared within and across different habitats. In most environments, HGT events were largely confined within the same habitat, indicating strong ecological structuring of phage–bacteria interactions (Fig. 3b). The highest within-habitat HGT events between phages and bacteria was observed in humans (408545), followed by freshwater, fish, mammals, and food habitats. In chicken, food, mammals, soil, sediment, and wastewater habitats, HGT events were detected exclusively within the same habitat, with no detectable cross-habitat transfer. These patterns indicate that habitat specificity strongly constrains phage-mediated toxin gene transfer across natural ecosystems. Among all habitats, the human environment was the major hotspot for toxin gene exchange. The highest cross-habitat HGT events was detected between humans and freshwater (20030). In habitats where cross-habitat transfer occurred, HGT events involving humans were consistently higher than those between other habitat pairs. For example, the cross-habitat HGT events between fish and freshwater (16) was substantially lower than that between fish and humans (360). Similarly, the HGT events between freshwater and phytoplankton (24) was lower than that between freshwater and humans (17 731; Fig. 3b). Such elevated connectivity between human and environmental habitats may reflect frequent environmental exchange processes, such as the release of human waste into aquatic systems.

The role of human-associated environments was further evaluated by calculating the average cross-habitat HGT events for each habitat. When HGT events involving humans were excluded, cross-habitat HGT events across habitats decreased by approximately one order of magnitude (Fig. 3c), further highlighting human environment as key hubs for toxin gene dissemination. Differences between within-habitat and average cross-habitat HGT events were also examined. In nine habitats, including humans, fish, food, and mammals, within-habitat HGT events exceeded the average cross-habitat events (Fig. 3c). In contrast, freshwater, ocean, laboratory, turtle and phytoplankton habitats initially showed more cross-habitat compared to within-habitat HGT events. However, after excluding HGT events involving humans, only three habitats (laboratory, ocean, and phytoplankton) retained more cross-habitat than within-habitat HGT events (Fig. 3c). Overall, systematic comparisons across habitats indicate that phage-mediated toxin gene transfer occurs predominantly within the same habitat, whereas human-associated environment was major hotspot facilitating toxin gene dissemination across ecosystems.

### Toxin-repression and anti-phage defense genes are negatively associated with phage-mediated HGT events

**Figure 4:**
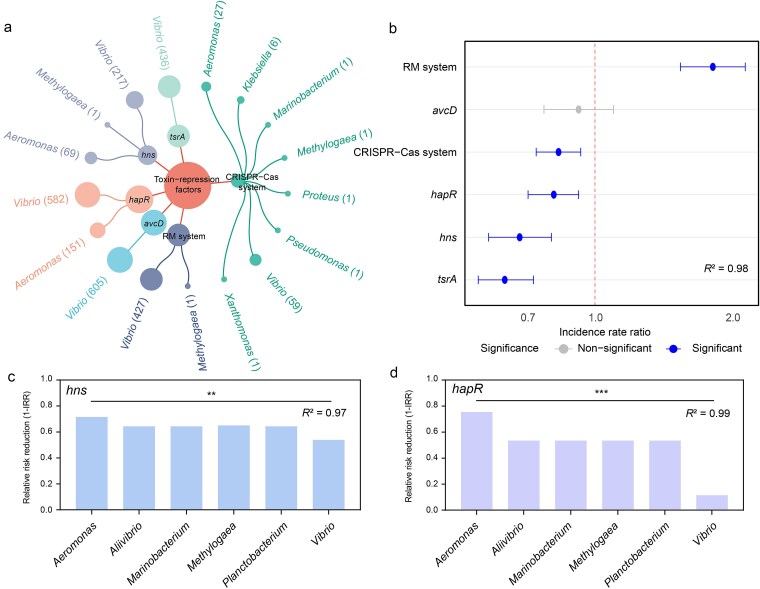
Anti-phage defense systems and toxin-repression genes constrain phage-mediated HGT events of toxin genes. a, Distribution of anti-phage defense systems (*avcD*, CRISPR–Cas, and restriction–modification (RM) systems) and toxin-repression genes (*hns, hapR*, and *tsrA*) across bacterial genera. b, Incidence rate ratios (IRRs) estimated using a generalized linear mixed model (GLMM; model 1) showing the association between defense or repression genes and toxin gene HGT events. Points indicate IRRs and bars denote 95.0% confidence intervals. Association was considered statistically significant when *P* < 0.05, and not significant when *P* > 0.05. An IRR of 1 indicates no effect on HGT events, IRR > 1 indicates an increased risk of HGT events, and IRR < 1 indicates a reduced risk. C, D, Relationship between the number of HGT events and the abundance of *hns* (c) and *hapR* (d) across bacterial genera, expressed with the relative risk reduction (1 − IRR). The value of 1 − IRR represents the estimated proportional reduction in predicted HGT events associated with each gene. Asterisks indicate statistical significance (*P* < 0.05, *P* < 0.01, *P* < 0.001).

Because bacteria possess multiple mechanisms to resist phage infection, the potential association between bacterial resistance mechanisms and phage-mediated HGT events of CTgenes was investigated. Eight bacterial genera, including *Vibrio, Aeromonas, Klebsiella, Marinobacterium, Methylogaea, Proteus, Pseudomonas*, and *Xanthomonas*, were identified as encoding anti-phage defense systems (Fig. 4a; Table S7). In contrast, toxin-repression genes were detected in three genera (*Vibrio, Aeromonas*, and *Methylogaea*). Generalized linear mixed-effects models (GLMMs) were applied to evaluate the effects of anti-phage defense systems (*avcD*, CRISPR–Cas, and R–M systems) and toxin-repression genes (*hns, hapR*, and *tsrA*) on phage-mediated HGT events involving toxin genes. In these models, the presence of defense and repression genes was treated as fixed effects, whereas bacterial genus was included with a random effect to account for potential genus-specific variation. This framework enabled assessment of whether the IRRs of these factors differed across bacterial genera and whether the elevated toxin gene HGT events observed in *Vibrio* could be associated with reduced prevalence of these resistance mechanisms.

In model 1, correlations between the number of defense and repression factors (*avcD*, CRISPR–Cas, RM systems, *hns, hapR*, and *tsrA*) and the number of phage-mediated toxin gene HGT events were evaluated. The model showed a high conditional *R*^2^ of 0.98, indicating that fixed effects and genus-level random effects together explained 98% of the variance in HGT events. Model stability was supported by five-fold cross-validation (RMSE = 363.4 ± 13.5) and by the absence of overdispersion or influential outliers (*P* > 0.05; Table S8). Toxin-repression genes were negatively associated with phage-mediated HGT events (Fig. 4b). The presence of *tsrA* was associated with an 36.8% reduction in predicted HGT events (IRR = 0.63, *P* < 0.001), whereas *hns* (IRR = 0.68, *P* < 0.001) and *hapR* (IRR = 0.81, *P* < 0.001) were associated with 32.0% and 19.2% reductions, respectively. A similar negative association was observed for the CRISPR–Cas system (IRR = 0.83, *P* < 0.001), corresponding to a 17.1% reduction in predicted HGT events. In contrast, R–M systems showed a positive association (IRR = 1.83, *P* < 0.001) with HGT events.

Genus-specific effects of toxin-repression genes were further evaluated using separate GLMMs (models 2–4). Predicted relative risk reduction (1 − IRR) was used to quantify the magnitude of inhibition of HGT events. Significant genus-specific differences were detected for *hns* (model 2) and *hapR* (model 3) (ANOVA, *P* < 0.05; Fig. 4c, d), whereas the model 4 for *tsrA* failed to converge. The conditional *R*^2^ values for models 2 and 3 were 0.97 and 0.99, respectively, indicating that toxin-repression genes and genus jointly explained > 97% of the variance in HGT events. The predicted relative risk reduction associated with *hns* and *hapR* was the lowest in *Vibrio* compared with other genera (Fig. 4c, d), indicating a weaker inhibitory association with HGT in *Vibrio*. Collectively, these results indicate that toxin-repression genes (*hns* and *hapR*) were negatively associated with phage-mediated toxin gene transfer and that this inhibitory relationship was weaker in *Vibrio* than in other bacterial genera.

### Validation of the inhibitory effects of toxin-repression genes *hns* and *hapR* on HGT efficiency and phage population dynamics

**Figure 5:**
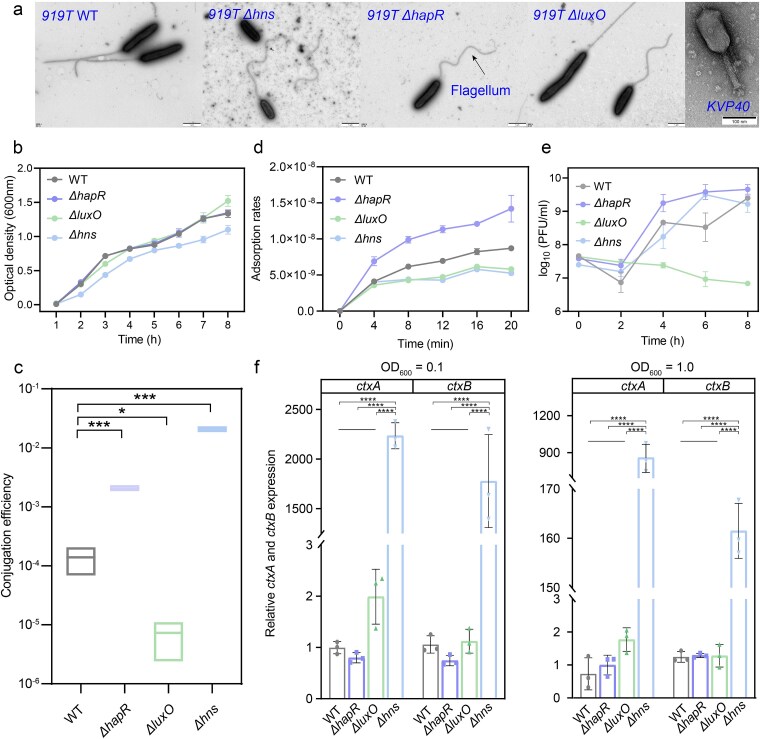
Toxin-repression genes *hapR* and *HNS* inhibit phage release in *Vibrio cholerae* 919T. a, Transmission electron microscopy (TEM) of *V. cholerae* 919T wild-type (WT), Δ*hns,* and QS mutant (Δ*hapR* and Δ*luxO*) strains with a scale bar of 1 μm. The TEM of KVP40 with a scale bar of 100 nm. b, The optical density (OD_600nm_) of *V. cholerae* 919T WT, Δ*hns*, Δ*hapR*, and Δ*luxO* strains was measured at 1h interval during 8 h incubation. c, The plasmid conjugation efficiency of *V. cholerae* 919T WT, Δ*hns*, Δ*hapR*, and Δ*luxO* strains. d, Adsorption assay of phage KVP40 on strain WT, Δ*hns*, Δ*hapR*, and Δ*luxO*. Phage adsorption rates were normalized by the initial bacterial concentration. Results are representative of three independent experimental assays. e, KVP40 particle density during KVP40 infection with *V. cholerae* 919T WT, Δ*hns*, Δ*hapR*, and Δ*luxO* was measured at 2h interval during 8 h incubation, plotted as the log_10_ of the percent of free phage in the cell-free spent supernatant. f, The relative mRNA expression levels of the cholera toxin genes (*ctxA* and *ctxB*) in *V. cholerae* 919 T WT, Δ*hns*, Δ*hapR*, and Δ*luxO* strains were measured at bacterial densities of OD_600nm_ = 0.1 and 1.0, with the housekeeping gene *recA* as the internal reference. Asterisks indicate statistical significance, where ^*, **, ***^, and “ns” denote *P* < 0.05, *P* < 0.01, *P* < 0.001, and *P* > 0.05, respectively.

Bacterial anti-phage defense systems are widely known to inhibit phage infection [37], while *hns* and *hapR* has been shown to play roles in phage resistance across multiple bacterial species, including *V. cholerae, V. anguillarum*, and *E. coli* [23, 31, 38–42]. Two toxin-repression genes, *hns* and *hapR*, were experimentally evaluated in *V. cholerae* El Tor 919 T for their effects on phage–bacterium interactions (including phage adsorption and phage progeny release) [43–46]. Because the QS regulator *luxO* represses *hapR* expression at low cell densities, *luxO* was also deleted to generate a *hapR*-overexpressing background. Three mutant strains (Δ*hns*, Δ*hapR*, and Δ*luxO*) were therefore constructed in the *V. cholerae* 919 T background. Deletion of *hns* markedly affected bacterial growth. Colonies of the Δ*hns* were visibly smaller than those of the WT (Fig. 5a). Consistently, optical density measurements showed significantly reduced growth of Δ*hns* relative to WT between 2 and 8 h (except at 5 h) (*P* < 0.001, one-way ANOVA), with OD_600_ = 1.0 reached at 6 h for WT, Δ*hapR*, and Δ*luxO*, but only at 8 h for Δ*hns* (Fig. 5b), indicating that *hns* deletion compromises bacterial growth. We also used plasmid conjugation assay to test if toxin-repression genes might constrain HGT at more general level. Compared to the WT strain, the Δ*hns* and Δ*hapR* mutants showed a significant increase in conjugation efficiency (*P* < 0.05, *t-*test; Fig. 5c), while the Δ*luxO* mutant showed a significant decrease in conjugation efficiency (*P* < 0.05, *t-*test; Fig. 5c). These results indicate that H-NS and HapR function as negative regulators of HGT. Because phage KVP40 exhibits a broad host range beyond the genus *Vibrio*, the adsorption and progeny release capacities of the four strains were further examined. Phage adsorption rate was significantly reduced in the Δ*hns* and Δ*luxO* mutants relative to WT at last time points (*P* < 0.05, one-way ANOVA; Fig. 5d), whereas Δ*hapR* displayed significantly higher adsorption rate than WT (*P* < 0.05; Fig. 5d). These results indicate that H-NS has a positive effect while HapR has a negative effect on KVP40 adsorption.

Phage progeny production following infection was also quantified. After 6 h of infection, both Δ*hapR* and Δ*hns* mutants produced significantly more phage particles than the WT strain (*P* < 0.05, one-way ANOVA; Fig. 5e), whereas the Δ*luxO* mutant produced fewer phage particles (*P* < 0.05; Fig. 5e). These results suggest that *hapR* and *hns* suppress phage release, whereas *luxO* promotes phage replication. Collectively, these findings indicate that toxin-repression genes reduce phage fitness by limiting adsorption (*hapR*) and repressing phage release (*hns* and *hapR*). Virulence-associated traits were further examined by assessing biofilm formation and expression of the CTgenes *ctxA* and *ctxB*. Biofilm formation was significantly reduced in Δ*luxO* (~2–3-fold decrease) relative to WT (*P* < 0.05, one-way ANOVA; Fig. S6), whereas Δ*hapR* exhibited significantly enhanced biofilm formation (*P* < 0.05; Fig. S6). No significant difference was observed in Δ*hns* (*P* > 0.05; Fig. S6). Expression of *ctxA* and *ctxB* was further quantified at low (OD_600_ = 0.1) and high (OD_600_ = 1.0) cell densities. Deletion of *hns* resulted in a significant increase in *ctxA* and *ctxB* expression at both cell densities (*P* < 0.001, one-way ANOVA; Fig. 5f), indicating that *hns* represses CTgene expression. In contrast, Δ*hapR* and Δ*luxO* showed no significant changes in toxin gene expression (*P* > 0.05; Fig. 5f). Together, these results demonstrate that toxin-repression genes modulate HGT efficiency, phage infection dynamics, and virulence traits in *V. cholerae*. Specifically, *hns* and *hapR* reduce phage fitness by limiting adsorption (*hapR*) and progeny release (*hns* and *hapR*), also attenuating pathogenicity through repression of toxin expression (*hns*) and biofilm formation (*hapR*).

## Discussion

Our results suggest that ecological context strongly shapes the dynamics of CTgene transfer. HGT events were consistently greater within habitats than between habitats, indicating that ecological compatibility between phages and their bacterial hosts facilitates gene exchange. Similar environmental conditions also share similar selective pressures, and high host densities likely promote stable phage–host interactions that increase opportunities for toxin gene transfer. This pattern is consistent with previous studies showing that ecological niches rather than geographic distances often structure global HGT networks in microbial communities [27, 47]. Because phages can disperse across regions through ocean circulation and human movement, ecological filtering within habitats may represent a key mechanism determining where toxin gene exchange occurs [2, 27]. The uneven distribution of genomic sequencing across regions can lead to an underestimation of the prevalence and diversity of CTgenes in high-burden but under-sequenced areas. Regions such as sub-Saharan Africa remain underrepresented in public genomic databases, limiting our understanding of local *V. cholerae* populations, CTXφ phages, and phage-mediated toxin gene evolution. To mitigate this, we applied a case-weighted genomic abundance framework using reported cholera incidence, providing a more epidemiologically informed view of global toxin gene distribution. Nevertheless, dedicated genomic surveillance in resource-limited, cholera-endemic regions, combined with metagenomic sequencing, is essential to obtain a more accurate and equitable representation of the global CTgene landscape.

Temporal analysis revealed relatively low phage-mediated HGT events involving toxin genes (*ace, ctxA, ctxB*, and *zot*) between 1940 and 2000 (Fig. S5), suggesting that during early stages of the seventh cholera pandemic, *V. cholerae* evolution was primarily clonal and driven by point mutations [48]. In contrast, HGT events peaked between 2010 and 2020, coinciding with rapid lineage turnover and global spread during the third pandemic wave (Fig. S5). Increasing global human mobility likely accelerated mixing of pathogenic strains and phages, exemplified by the introduction of *V. cholerae* from Nepal into Haiti in 2010 [49]. Localized cholera outbreaks in the twenty-first century, including Dhaka (2006), Zimbabwe (2008), Haiti (2010), and Kenya (2010), may have further promoted conditions favorable for phage-mediated toxin gene transfer. Within the analyzed dataset, toxin-containing bacterial genomes were most frequently recovered from human-derived samples, followed by freshwater environments and fish (Fig. 2b). Human- and freshwater-associated genomes were predominantly affiliated with *Vibrio*, whereas fish-associated genomes were more frequently affiliated with *Aeromonas* (Fig. 2b). Toxin-encoded phages followed a similar pattern, with the majority derived from human samples and predominantly classified within the *Inoviridae* family (Fig. 2d, Table S2).

Bacterial defense systems, including CRISPR–Cas, R–M systems, and *avcD*, were negatively associated with phage-mediated toxin gene transfer [44, 50, 51]. Additional mechanisms, such as adsorption inhibition, toxin–antitoxin modules, and abortive infection pathways, likely also contribute to limiting HGT [52], and future studies should explore a broader range of anti-phage defenses in both *Vibrio* and non-*Vibrio* bacteria. Toxin-repression genes (*hns, hapR*, and *tsrA*) also significantly constrained phage-mediated toxin gene transfer. In non-*Vibrio* genera, H-NS and HapR had stronger inhibitory effects than in *Vibrio*, which may explain the lower toxin gene transfer events among non-*Vibrio* bacteria. We found that phage genomes infecting non-*Vibrio* bacteria exhibit significantly lower AT content compared to those infecting *Vibrio* bacteria (mean AT content of phage genomes: 49.1% ± 1.8% *v.s.* 54.9% ± 0.7%, *P* < 0.001, *t-*test; Table S9). The reduced AT content in phage genomes may reduce H-NS binding probability at excisionase gene binding sites, leading to accelerated prophage excision in non-*Vibrio* bacteria. Rapid phage excision shortens the time window for phage-bacterial genome recombination during the lysogenic phase, thereby reducing opportunities for HGT events. However, the inhibitory effect of *hns* on phage excision in non-*Vibrio* and *Vibrio* bacterial genomes needs more study and direct experimental comparisons.

H-NS acts as a xenogeneic silencer targeting AT-rich DNA and represses prophage excision in several bacterial species [30, 38, 53–56]. Experimentally, H-NS inhibited the replication of the lytic phage KVP40 in *V. cholerae* (Fig. 5e), supporting its role in promoting phage latency. While this is not direct evidence for reduced phage-mediated HGT, phage burst size has been shown to positively correlate with phage transduction efficiency [57]. Therefore, reduced phage release may also limit phage transduction. In addition, the presence of *hns* genes indirectly regulates phage CTXφ infection by causing the downregulation of genes encoding CT and TCP in *V. cholerae* [58, 59]. HapR, a QS regulator, also reduced phage adsorption and release, consistent with its accumulation under high-cell-density conditions in the gut and its reported role in downregulating TCP and CTexpression [18, 46, 60]. After normalization of adsorption rates by initial bacterial density, the Δ*luxO* exhibited reduced, whereas Δ*hapR* displayed higher phage adsorption rate compared with WT (Fig. 5d). This observation is consistent with previous reports that QS activation can decrease phage susceptibility of bacterial cell through reduced phage receptor availability [39, 42]. These toxin-repression genes (*hns* and *hapR*) and defense systems identified as determinants of phage-mediated HGT are not independent of one another. For example, CRISPR-Cas systems are subject to complex regulation by QS or H-NS across multiple species, including *Pseudomonas aeruginosa, Staphylococcus aureus*, and *Escherichia coli* [61–63]. However, the directionality of QS and H-NS mediated regulation of CRISPR-Cas systems remains unclear, with both positive and negative roles previously reported [61–64]. The CBASS abortive infection system in *V. cholerae* can also be regulated by the QS regulator HapR at high cell densities [20]. In summary, beyond possible direct effects on phage replication, toxin-repression genes may also influence phage-mediated HGT indirectly through modulation of defense system activity. Future studies should investigate how toxin-repression genes influence specific defense systems, and whether their effects are conserved across diverse *V. cholerae* lineages and other pathogenic bacteria.

KVP40 is a T4-like lytic phage, whereas CTXφ is a filamentous phage that is typically released through a chronic, non-lytic mechanism without immediate host death. The observed inhibitory effect of H-NS and HapR on KVP40 adsorption and release should hence be interpreted primarily as evidence for host regulatory effects on lytic phage susceptibility and general phage production capacity, which could also affect regulation of filamentous phage release. Moreover, H-NS contributes to phage defense by linking virulence regulation with cellular anti-phage systems, highlighting a convergence between global regulatory networks and specialized immunity in *V. cholerae*. Consistent with recent reports, systems such as the DarTG toxin–antitoxin module, Type IV restriction system targeting modified cytosines, and QS-activated cyclic oligonucleotide based anti-phage signaling system (CBASS) illustrate how *V. cholerae* coordinates multiple layers of defense system to limit phage predation and maintaining environmental adaptability [20, 65].

Although acquisition of toxin genes such as *ctxA* and *ctxB* may influence host–pathogen interactions by altering intestinal physiology and nutrient availability, we emphasize that our data do not directly demonstrate a causal effect of HGT on nutrient acquisition. Nonetheless, these genes likely provide a selective advantage for toxigenic *V. cholerae* strains during infection [66]. Together, our results provide a conceptual framework in which ecological context, phage–host interactions, and bacterial regulatory systems jointly shape the global dynamics of CTgene transfer. This framework highlights how phage-mediated HGT can facilitate the spread of virulence factors, also identifying intrinsic bacterial mechanisms that constrain these transfers. Integrating genomic, ecological, and experimental approaches will be essential for predicting how phage-mediated gene transfer influences the emergence and evolution of pathogenic *Vibrio* populations.

## Conclusions

Global genomic analyses revealed that *V. cholerae* isolates and phages carrying CTgenes are more frequently detected in human-associated habitats than in natural environments, suggesting that environmental reservoirs remain underexplored. Extensive HGT of CTgenes between phages and bacteria was observed, highlighting the central role of phages in disseminating these genes across both human-associated and natural ecosystems. HGT events were greater within the same habitat than between different habitats, indicating that local phage–bacteria interactions may facilitate habitat-specific adaptation and gene exchange. Statistical analyses demonstrated that bacterial defense mechanisms are strongly associated with reduced phage-mediated toxin gene transfer, with CRISPR–Cas systems and toxin-repression genes showing negative correlations with HGT events. *hns* and *hapR* genes encoded by non-*Vibrio* bacteria exhibited stronger inhibitory effects on phage-mediated toxin gene transfer than those encoded by *Vibrio*. Experimental validation confirmed that H-NS and HapR suppress phage release, thereby limiting environmental phage abundance and toxin gene transfer. Collectively, these findings emphasize the pivotal role of phages in spreading CTgenes and the counteracting mechanisms employed by bacteria. Further studies are warranted to determine how toxin-repression genes across diverse bacterial species modulate phage-mediated HGT and influence cross-species dissemination of cholera toxin.

## Supplementary Material

Supplementary_material_wrag139

## Data Availability

Phage sequences carrying the cholera toxin genes (*ctxA, ctxB, ace*, and *zot*) were retrieved from the publicly available IMG/VR database (version 4.1) (https://img.jgi.doe.gov/cgi-bin/vr/main.cgi). Genomes of *Vibrio cholerae* and other bacteria containing toxin genes were identified by searching the EnteroBase (https://enterobase.warwick.ac.uk/species/index/vibrio) and the NCBI RefSeq database (https://www.ncbi.nlm.nih.gov/refseq/). All bacterial genomes analyzed in this study are publicly available in the Figshare repository (10.6084/m9.figshare.24800075).
