# Supplementary material for: Global transmission and distribution of phage-encoded cholera toxin genes constrained by toxin-repression genes and anti-phage defense systems
Source: ISME J. 2026 Jun 11;20(1):wrag139. doi: 10.1093/ismejo/wrag139 (PMC13310137; doi:10.1093/ismejo/wrag139)
Supplement: Supplementary_material_wrag139 [file supplementary_material_wrag139.zip › supplementary_Figures_260508_wrag139.docx]

**
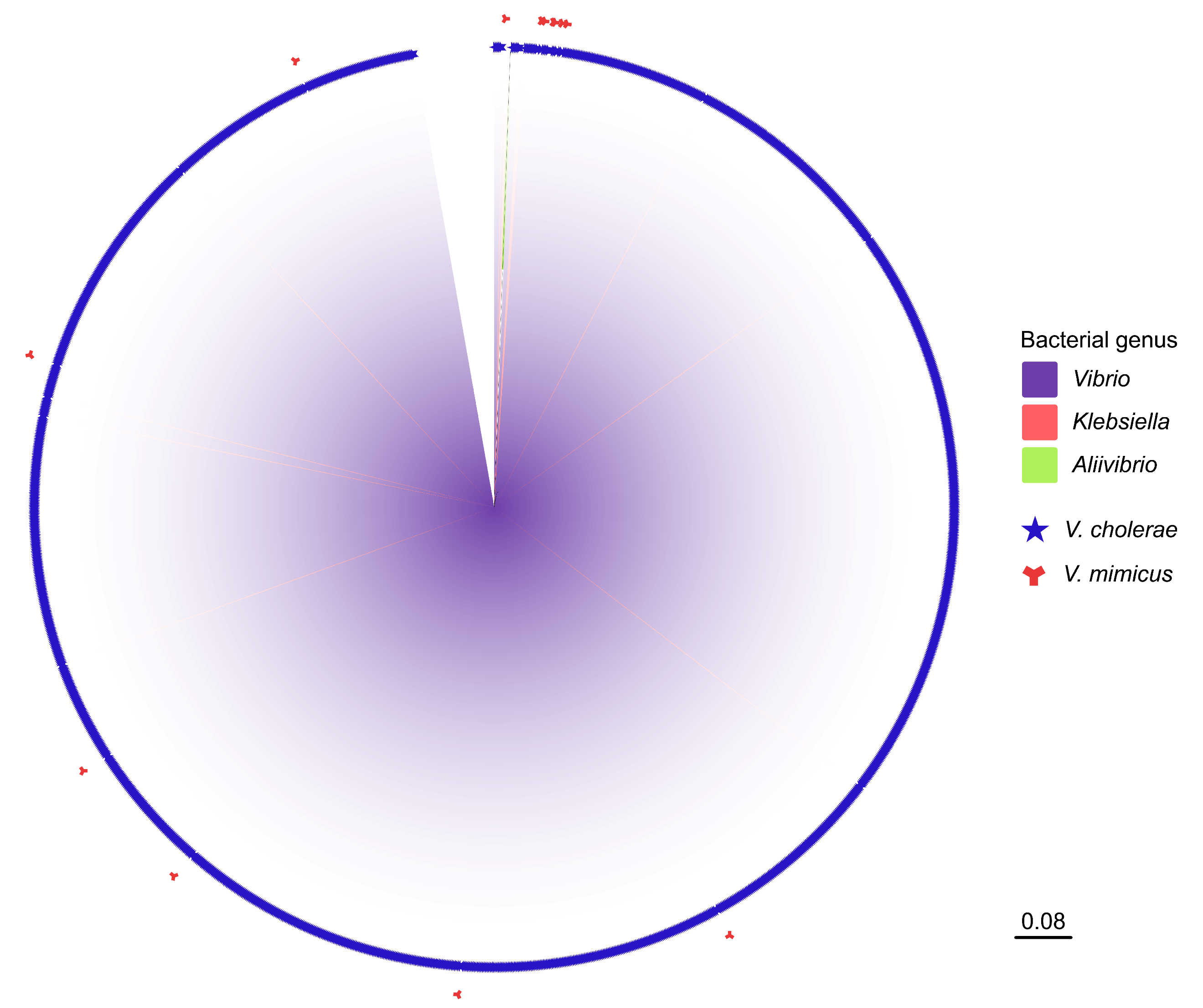
**

**Fig.S1** Phylogenetic tree of the Ace toxin protein in bacterial genomes. Different shadow colors represent different bacterial genera. Bacterial species belonging to the *Vibrio* are marked using different shapes.

**
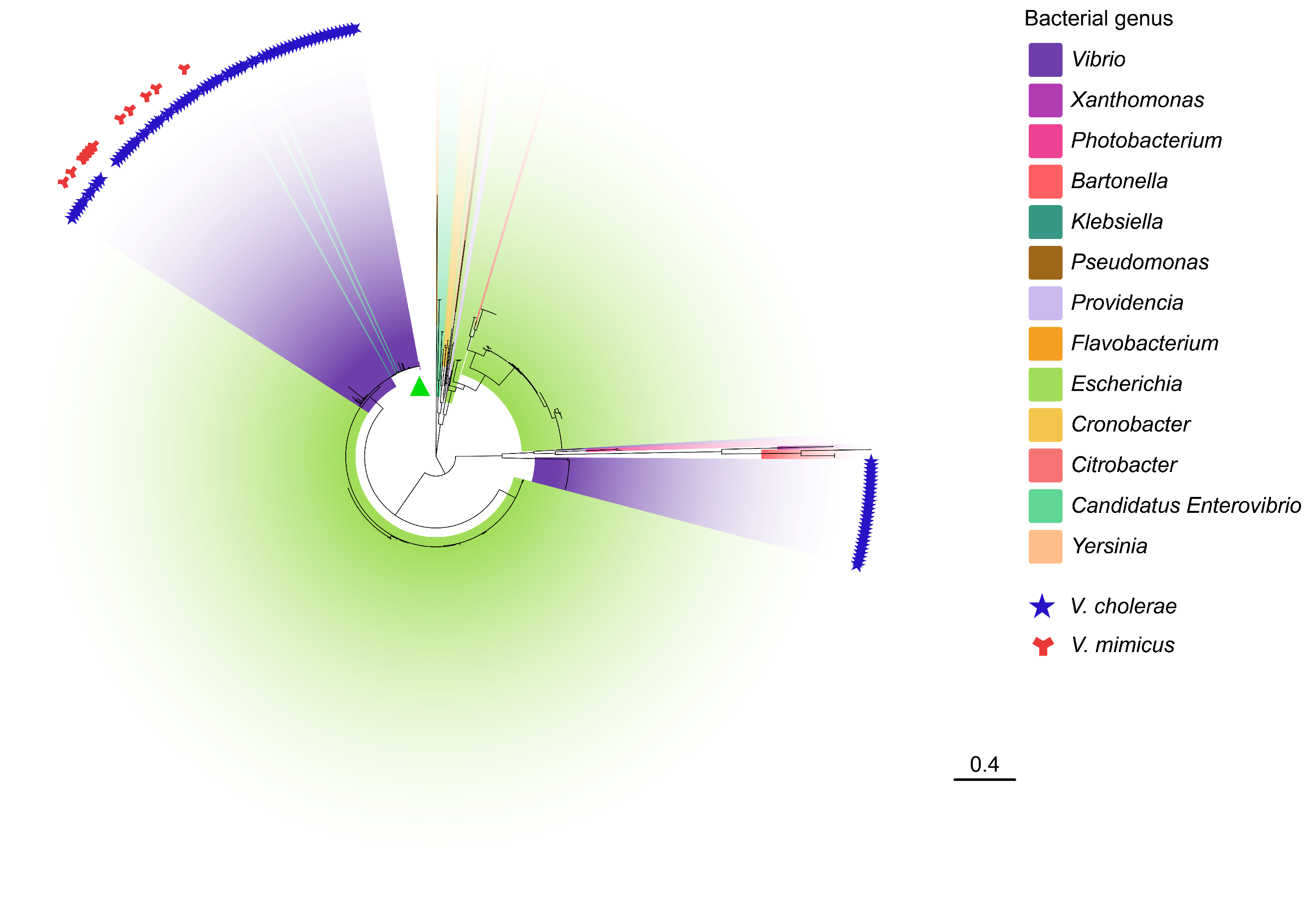
**

**Fig.S2** Phylogenetic tree of the CtxA toxin protein in bacterial genomes. Different shadow colors represent different bacterial genera. The green triangle marks the collapsed CtxA branch of *V. cholerae*. Bacterial species belonging to the *Vibrio* are marked using different shapes.


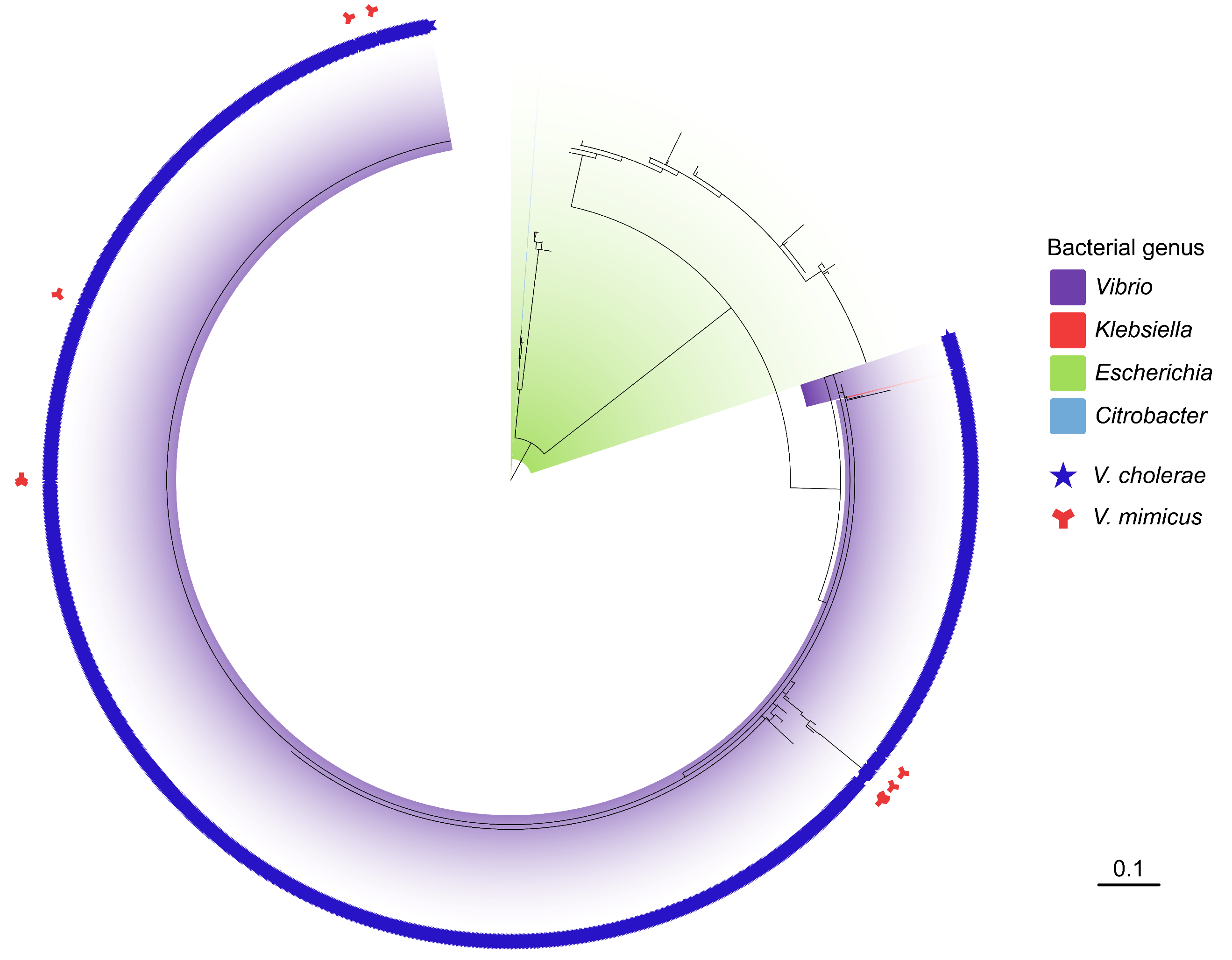


**Fig.S3** Phylogenetic tree of the CtxB toxin protein in bacterial genomes. Different shadow colors represent different bacterial genera. Bacterial species belonging to the *Vibrio* are marked using different shapes.


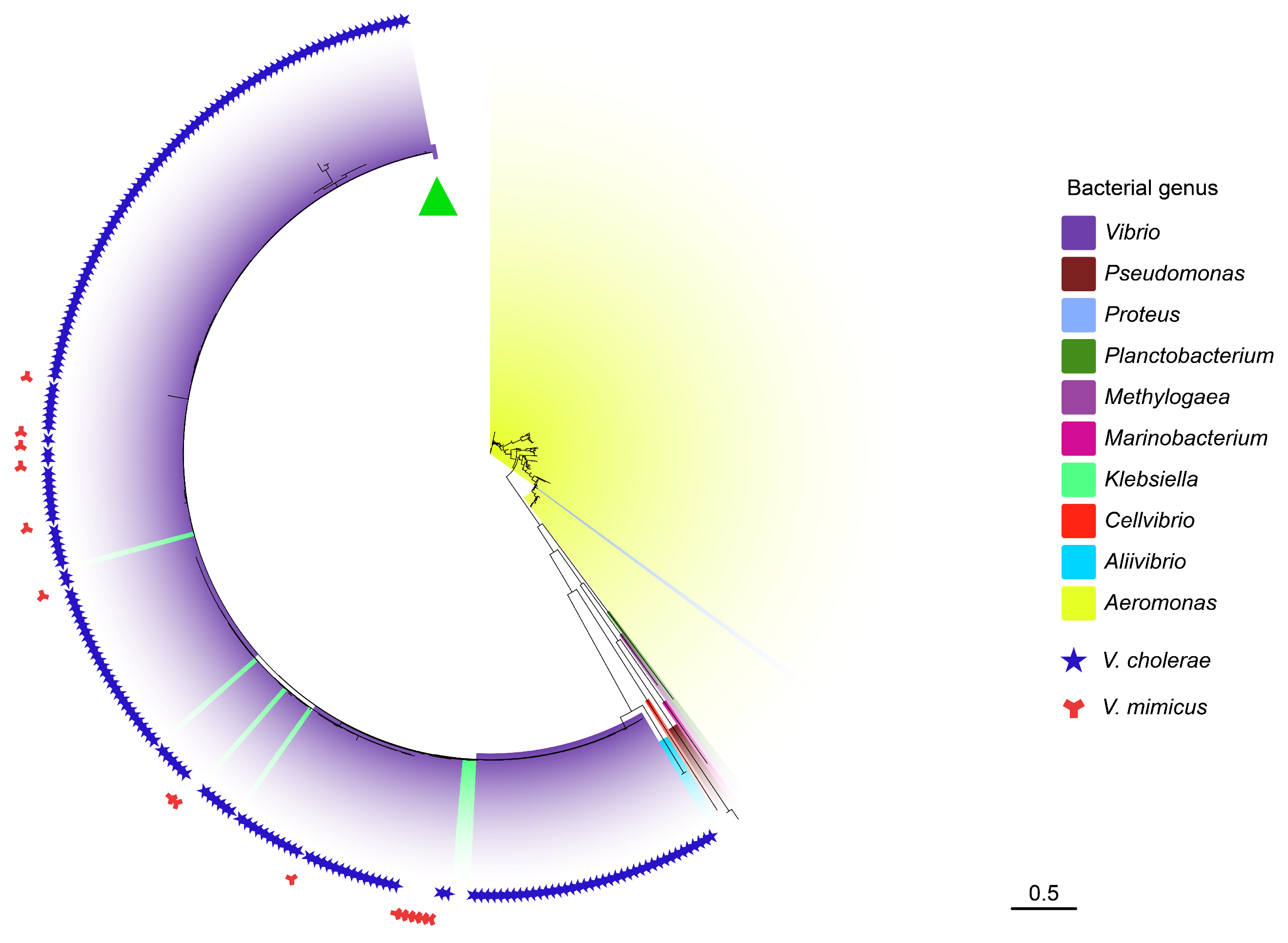


**Fig.S4** Phylogenetic tree of the Zot toxin protein in bacterial genomes. Different shadow colors represent different bacterial genera. The green triangle marks the collapsed Zot branch of *V. cholerae*. Bacterial species belonging to the *Vibrio* are marked using different shapes.


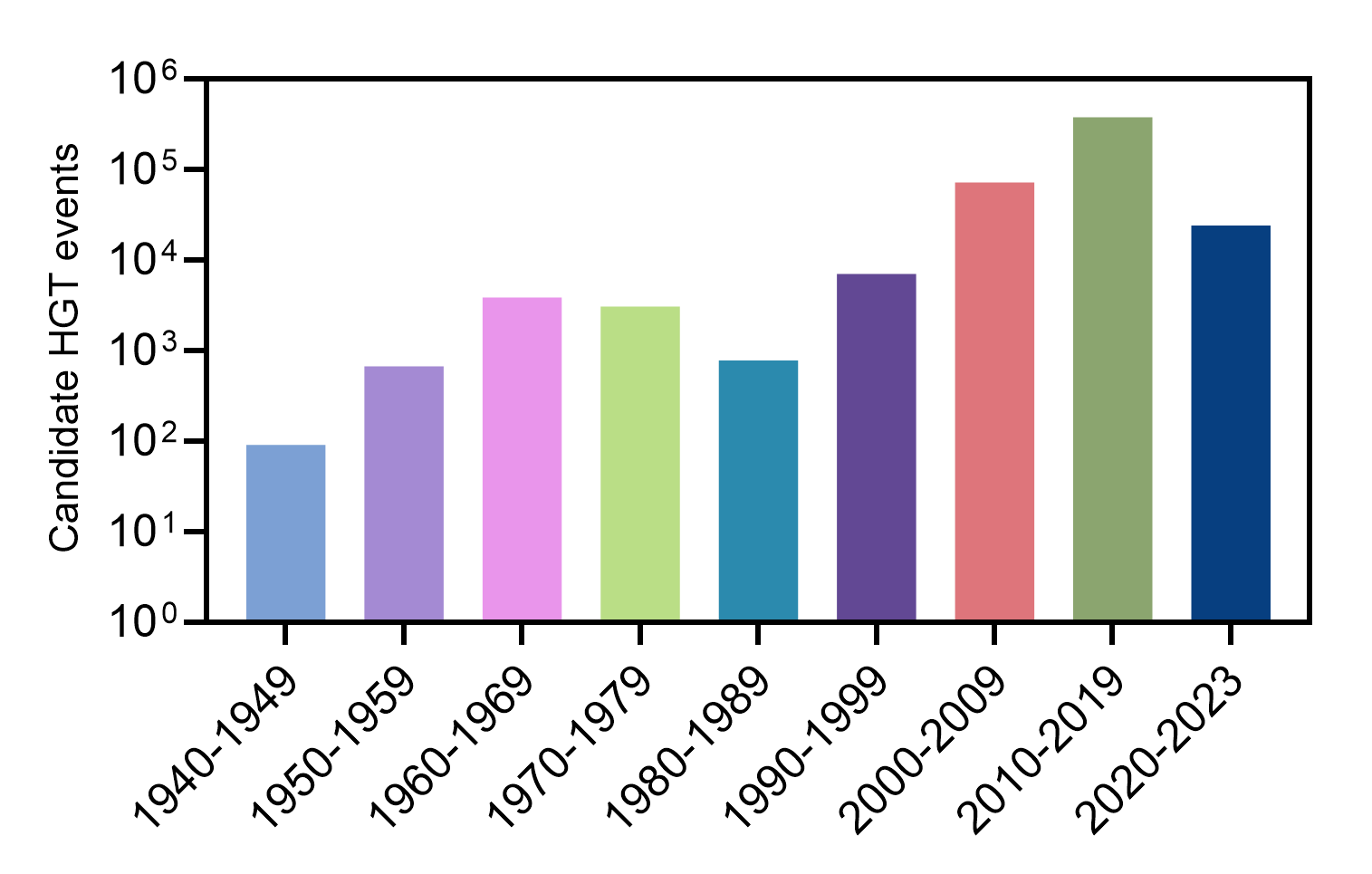


**Fig.S5** The *Inoviridae* phage-mediated candidate HGT events of toxin genes across different years.


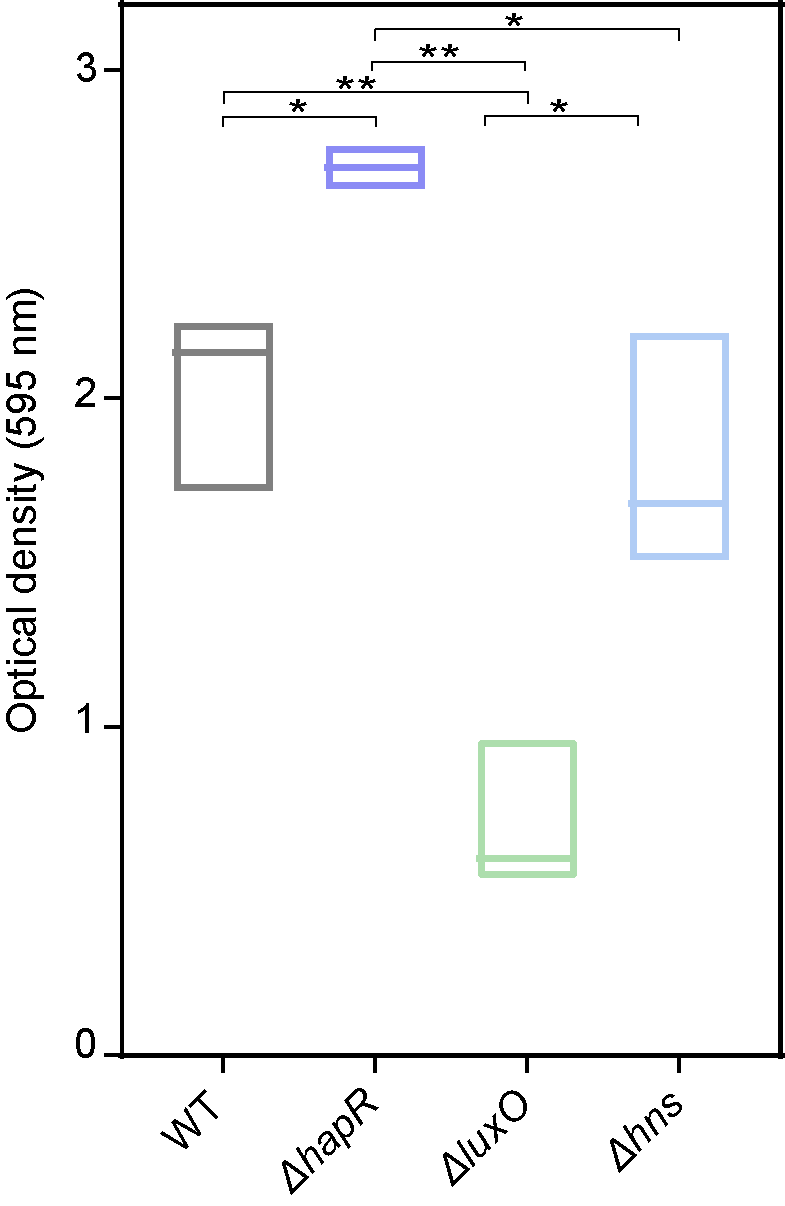


**Fig.S6** The biofilm formation (OD_595nm_) characteristics of *V. cholerae* 919T WT, *Δhns*, *ΔhapR*, and *ΔluxO* strains.
